# IgG4-related disease of pulmonary artery causing pulmonary hypertension

**DOI:** 10.1097/MD.0000000000010698

**Published:** 2018-05-18

**Authors:** Hui Deng, Sheng Zhao, Yunlong Yue, Yong Liu, Yali Xu, Jin Qian, Xiaorong Ma, Peiliang Gao, Xiaoyan Yao, Xin Jiang, Xiqi Xu, Zhicheng Jing, Yong Wang, Lei Pan, Xinying Xue

**Affiliations:** a Department of Respiratory Disease; b Department of Cardiology; c Department of MRI; d Department of Ultrasound, Beijing Shijitan Hospital, Capital Medical University; e Thrombosis and Vascular Medicine Center, Vascular Biology Research Unit in State Key Lab of Cardiovascular Disease, Fuwai Hospital & National Center for Cardiovascular Disease, Peking Union Medical College & Chinese Academy of Medical Sciences, Beijing, China.

**Keywords:** IgG4-related disease, positron emission tomography/computed tomography, pulmonary hypertension

## Abstract

IgG4-related disease (IgG4-RD) is recognized as an immune-mediated condition with pathology features of lymphoplasmacytic infiltrate, storiform fibrosis, and obliterative phlebitis, accompanied with or without elevated serum IgG4 concentrations. However, few of pulmonary artery IgG4-RD causing pulmonary hypertension (PH) was reported.

The medical records of 3 patients with pulmonary artery IgG4-RD inducing PH were analyzed retrospectively.

Imaging findings demonstrated that the lesions of 3 patients located in pulmonary artery, which were initially diagnosed as pulmonary thrombus or malignant tumor. Computed tomography pulmonary angiography (CTPA), ultrasonic cardiogram, and positron emission tomography/computed tomography (PET/CT) didn’t support the diagnosis of pulmonary thrombus or malignant tumor. Right heart catheterization (RHC) showed definite PH. Biopsy by right heart catheterization in 2 patients or pneumonectomy in 1 patient confirmed the diagnosis as IgG4-RD. Treated with glucocorticoids and cyclophosphamide or rituximab, 2 patients’ IgG4 concentrations declined sharply and the lesions shrunk gradually. Another patient treated with glucocorticoids died of heart failure.

IgG4-RD involved pulmonary artery causing PH was rare. A high index of awareness of this disease is required for early diagnosis and treatment. PET/CT might be a valuable approach to distinguish pulmonary artery IgG4-RD from pulmonary thrombus and malignant tumor.

## 1 Introduction

IgG4-related disease (IgG4-RD), involved single or multiple organs, is known as an immune-mediated disorder with typical histopathologic features, with or without elevated serum IgG4.^[1]^ The disease was recognized as a kind of systemic condition in 2003.^[2,3]^ Up to now, the available epidemiologic data, mainly derived from Japanese cohorts, estimated the annual incidence of IgG4-RD is 0.28 to 1.08/100,000.^[4,5]^ IgG4-RD has been reported involved in nearly every organ, including orbits and periorbital tissues, ears, nose, sinuses, salivary glands, meninges, pituitary, lymph nodes, thyroid glands, lungs, aorta, retroperitoneum, kidneys, pancreas, biliary trees, livers, skin, and prostates.^[6–10]^ IgG4-RD usually shows a favorable response to glucocorticoids therapy. However, published literature on pulmonary artery IgG4-RD causing pulmonary hypertension (PH) is limited. In this study, we aim to improve awareness of the clinical and imaging manifestations of pulmonary artery IgG4-RD causing PH to avoid a delay in diagnosis or misdiagnosis.

### 1.1 Patients and methods

Three patients were diagnosed as pulmonary artery IgG4-RD which initially was misdiagnosed as pulmonary thrombus or malignant tumor. The diagnosis of this disease was based on biopsy by right heart catheterizationin in 2 patients and pneumonectomy in 1 patient. We retrospectively analyzed these cases by reviewing these patients’ medical records, laboratory findings, radiological findings and pathological changes, therapeutical strategies, and following times. The Ethics Board in our hospital (Beijing Shijitan Hospital, Capital Medical University) approved our study and all patients provided written informed consent.

## 2 Results

**Figure 1:**
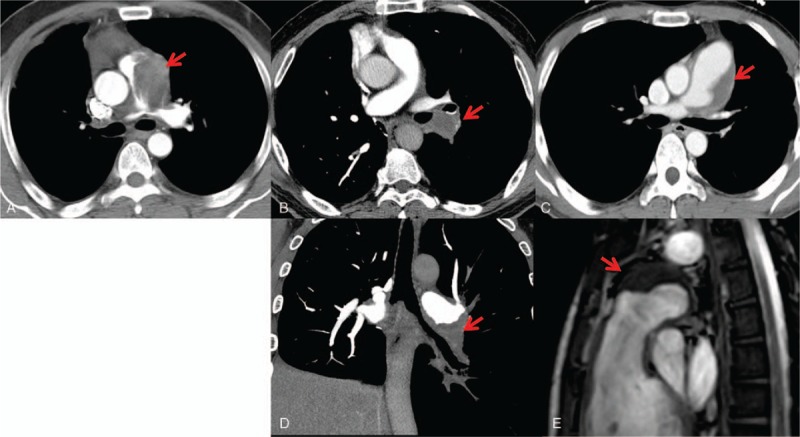
Imaging features of pulmonary artery IgG4-RD. Computed tomography pulmonary angiography (CTPA) demonstrated these lesions diagnosis of pulmonary artery IgG4-RD located in pulmonary artery (A: patient 1, B/D: patient 2, C/E: patient 3), whose lesions were initially diagnosed as pulmonary thrombus or malignant tumor. IgG4-RD = IgG4-related disease.

**Figure 2:**
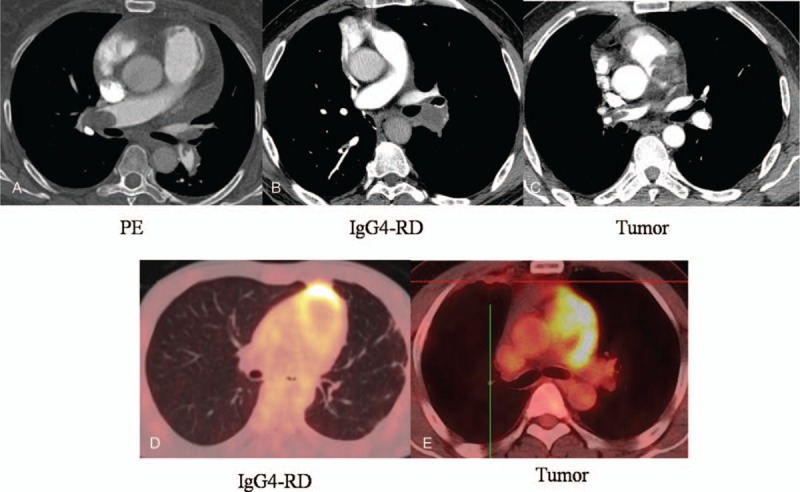
Imaging features to identify pulmonary artery IgG4-RD from PE and tumor. CTPA showed that the lesions (A and B) were not enhanced and the lesion (C) was obviously elevated. PET-CT demonstrated slight SUV intake (2.4) in the lesion (D), but obviously SUV intake (15.3) in the tumor lesion (E). CTPA = computed tomography pulmonary angiography, IgG4-RD = IgG4-related disease, PET/CT = positron emission tomography/computed tomography, SUV = standard uptake value.

**Figure 3:**
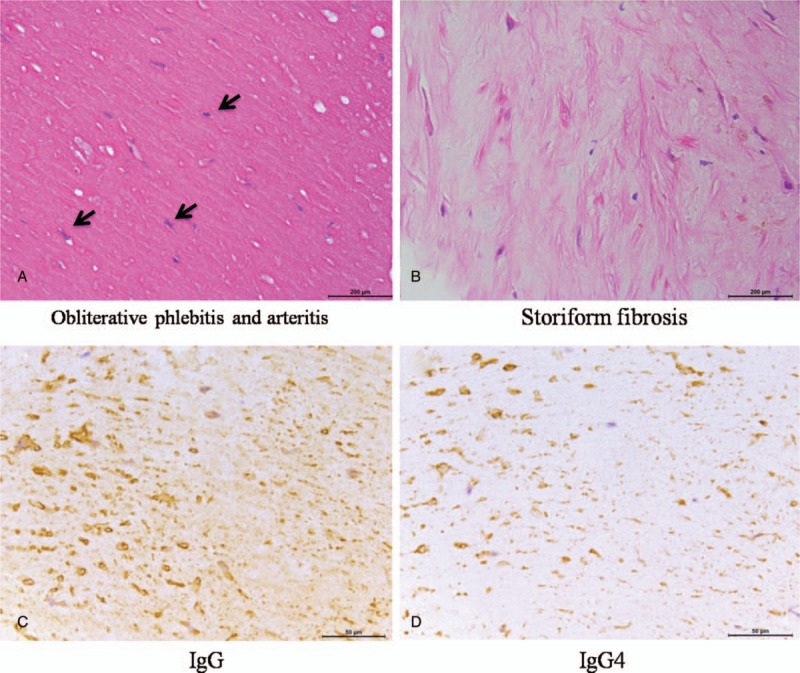
Pathological features of pulmonary artery IgG4-RD. Typical histopathologic features in patient 3, such as obliterative phlebitis and arteritis (A, ×100), storiform fibrosis (B, ×100) were demonstrated. Immunohistochemistry showed numerous IgG-reactive plasma cells are apparent (C, ×100). A significant proportion (46.38%) of the plasma cells also demonstrate reactivity with antibodies directed against IgG4 (D, ×100). IgG4-RD = IgG4-related disease.

The major clinical and auxiliary examination data of 3 patients (men) with pulmonary artery IgG4-RD were shown in Table 1. Their age ranged from 32 years to 52 years. There were 2 patients with short breath after activity and 1 with symptoms of cough, sputum, blood in phlegm, and hemoptysis. There was 1 patient with tuberculosis history, 1 with pneumonectomy as right granuloma lesion involved with pulmonary artery (PA), and 1 with no history. They had no travel history outside the state, and were not taking any medications before their admission to the hospital. Their parents and immediate family were healthy. Computed tomography pulmonary angiography (CTPA) and ultrasonic cardiogram or magnetic resonance imaging (MRI) demonstrated that the lesions of 3 patients located in PA, and all lesions were initially diagnosed as pulmonary thrombus or malignant tumor (Fig. 1). However, these indications, including fast blood stream at the location, elevated IgG4 serum concentrations, lesions without being enhanced by CTPA and lesions with slight standard uptake value (SUV) intake by positron emission tomography/computed tomography (PET/CT), did not support common pulmonary thrombus or malignant tumor (Fig. 2). Right heart catheterization biopsy in patient 1 and 3 and pneumonectomy biopsy in patient 2 finally confirmed the diagnosis as IgG4-RD. These lesions had typical histopathologic features (Fig. 3) including a rich lymphoplasmacytic, infiltrate, storiform fibrosis, obliterative phlebitis, and arteritis. Immunohistochemistry showed CD138 (+), IgG (+), IgG4 (+), IgG4/IgG (58.2%) in patient 1, AE1/AE3 (+), CD3 (+), CD20 (+), CD38 (+), IgG4/IgG20%, IgG4 (30/HPF) in patient 2, and CD20 (+), CD37 (+), CD38 (+), IgG4/IgG (46.38%) in patient 3.

**Table 1:**
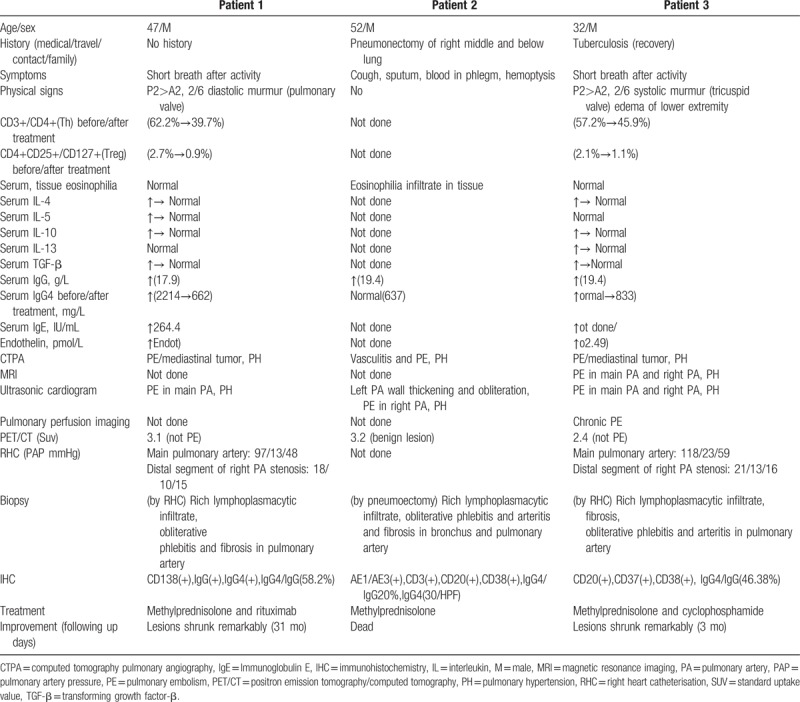
Clinical and auxiliary examination information of 3 patients with pulmonary artery IgG4-RD.

**Figure 4:**
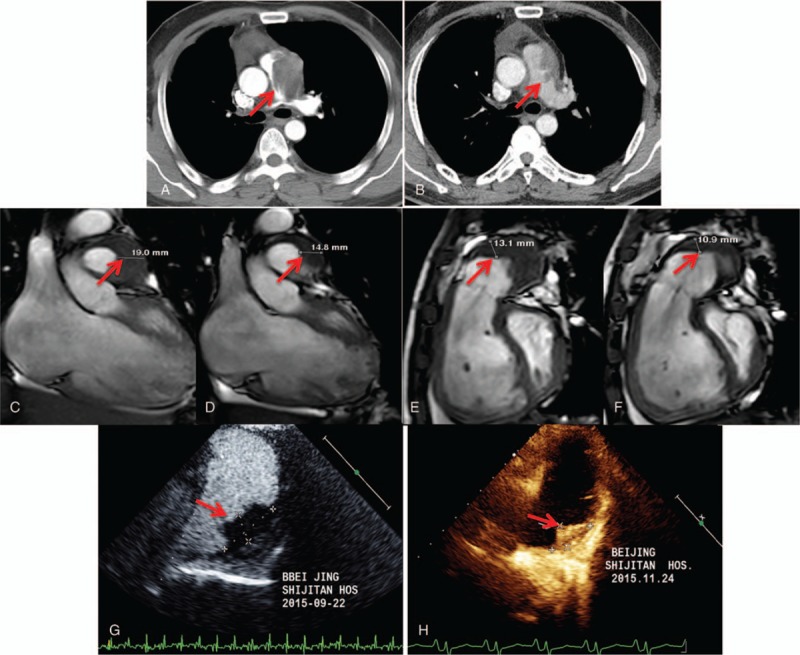
Imaging changes of before and after treatment. During 31 months follow-up, pulmonary CT demonstrated the lesion of pulmonary artery IgG4-RD in patient 1 shrunk obviously (A: before treatment, B: after treatment). During 3 months follow-up, MRI (C and E: before treatment, D and F: after treatment) and ultrasonic cardiogram (G: before treatment, H: after treatment) showed the lesion of pulmonary artery IgG4-RD also shrunk obviously in patient 3. CT = computed tomography; IgG4-RD = IgG4-related disease; MRI = magnetic resonance imaging.

Our initial management for these patients was to use glucocorticoids and cyclophosphamide for patient 3, glucocorticoids for patient 2, and glucocorticoids and rituximab for patient 1 as described in Table 1. During the period of follow-up after treatment, the lesions of pulmonary artery in patient 1 and 3 shrunk remarkably (Fig. 4), while patient 2 died of heart failure in following up.

Before treatment, expression levels of serum cytokines in patient 1 and 3, including interleukin-4, interleukin-5, interleukin-10, interleukin-13, transforming growth factor-β (TGF-β), immunoglobulin E, IgG4, and endothelin were substantially higher than that in healthy person (except for IL-5 in patient 3 and IL-13 in patient 1). After treatment, the serum IgG4 and ILs returned to normal. Before treatment, flow cytometry showed that CD3+/CD4+(Th) and CD4+CD25+/CD127+(Treg) were obviously high, and after treatment, these lymphocytes reduced obviously.

## 3 Discussion

Pulmonary artery IgG4-RD inducing PH is a rare form of IgG4-RD and might become another unnegligible cause of PH. IgG4-RD can involve virtually any organ. Besides the most frequently involved pancreas, biliary trees, and kidneys, it attacks large to medium-sized arteries extensively, such as abdominal aorta, thoracic aorta, iliac artery, coronary artery, renal artery. IgG4-RD involving pulmonary artery is rarely reported in published literature. From another angle, the lack of familiarity with its clinical and imaging manifestations may limit its diagnostic rate.

In this study, these 3 patients’ symptoms and physical examination were unremarkable, and all of them have no multi-organ involvement, which can be a vital clue for differential diagnosis. Besides that, elevated serum IgG4 concentrations can be a valuable hint leading us to a quick diagnosis. However, the serum IgG4 level is not a routine item of blood tests in current clinical practices. Furthermore, as a general conclusion, pathological diagnosis is the gold standard for diagnosing IgG4-RD, but it may be difficult to obtain biopsy specimen in IgG4-RD with pulmonary artery involvement in some cases, and this main method for definite diagnosis make little sense in early diagnostic procedures. So the delay in diagnosis or even misdiagnosis is hardly avoidable. Back to this study, we find that imaging examination played a crucial role in the diagnosis process, and may be an alternative method for the diagnosis.

CTPA and ultrasonic cardiogram are important diagnostic approaches to pulmonary vascular lesion. However, it is difficult to identify pulmonary artery IgG4-related lesions from malignant tumors and pulmonary thrombus. Recently, several case reports have indicated PET/CT scan might be a valuable approach to diagnosis of some IgG4-RD.^[11–15]^ In our study, PET/CT is also considered as an effective method to distinguish IgG4-RD from malignant tumors and pulmonary thrombus. The malignant tumor showed higher SUV intake while pulmonary thrombus showed no SUV intake. Proven useful in diagnosing the pulmonary artery IgG4-RD, these image characteristics of PET/CT should be explored and validated in further study and may be updated into the consensus diagnostic criteria for IgG4-RD in the future.

PH from the 5th World Symposium at Nice, France is divided to 5 classifications, including pulmonary arterial hypertension, pulmonary hypertension due to left heart disease, pulmonary hypertension due to lung diseases and/or hypoxemia, chronic thromboembolic pulmonary hypertension and pulmonary hypertension with unclear and/or multifactorial mechanisms.^[16]^ The guideline didn’t involve pulmonary artery IgG4-RD causing PH. In our study, we show 3 patients with pulmonary artery IgG4-RD causing PH, which presents one new contributory factor of the cause of PH.

The glucocorticoids is the first-line, standard approach for most patients with IgG4-RD.^[17,18]^ A Japanese study showed complete remissions in only 61% of IgG4-RD patients in 1 year following up.^[19]^ The response to glucocorticoids is different according to affected organs and the degree of fibrosis.^[2]^ Retroperitoneal fibrosis, sclerosing mesenteritis, and fibrosing mediastinitis are less effective to glucocorticoids. In our study, early treatment of glucocoricoids combined application conventional steroid-sparing agents or rituximab lead to good clinical outcomes, which may generate clues for further medication study. In addition, the serum IgG4 and cytokines concentration may become some good biomarkers for IgG4-RD improvement, which should be explored and validated in further study. The radiological assessment to pulmonary artery lesions by CTPA, MRI, and ultrasonic cardiogram is also an appropriate evaluation method for disease process.

**Figure 5:**
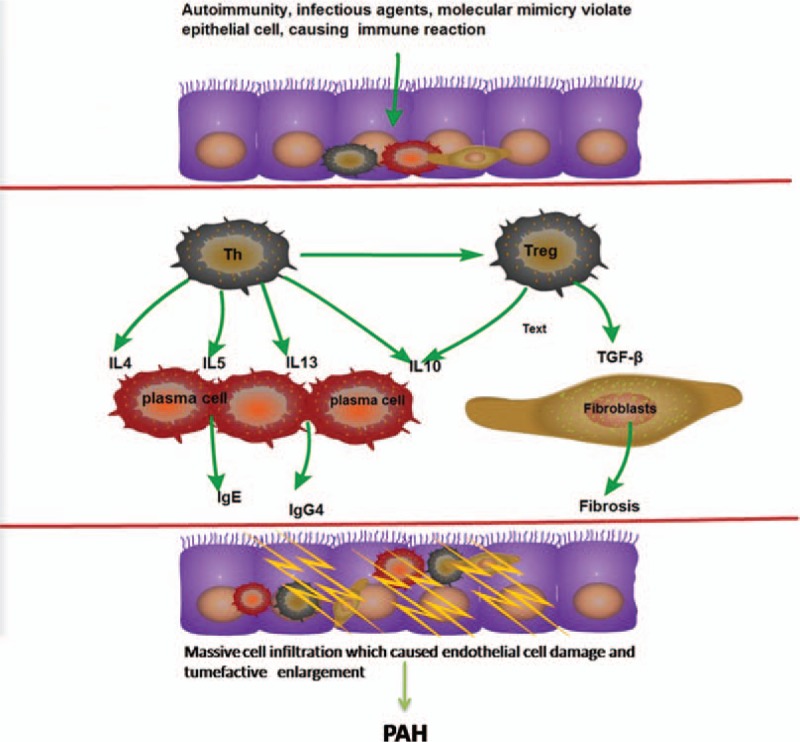
Probable pathogenetic mechanism in pulmonary artery IgG4-RD causing PH according to the changes of serum cytokines in Table 1. After potential triggers violate epithelial cell of pulmonary artery, Th2-cell responses are activated at affected sites, and then Treg cells are also activated. They together produce IL-4, IL-5, IL-10, IL-13, TGF-β which inducing plasma cell that produce high IgG4 and fibrosis. Elevated IgG4 and fibrosis contribute to destructive inflammation of endothelial cells and formation of granuloma. The granuloma of pulmonary artery and lots of endothelin cause PH at last. IgG4-RD = IgG4-related disease, PH = pulmonary hypertension, TGF-β = transforming growth factor-β.

**Figure 6:**
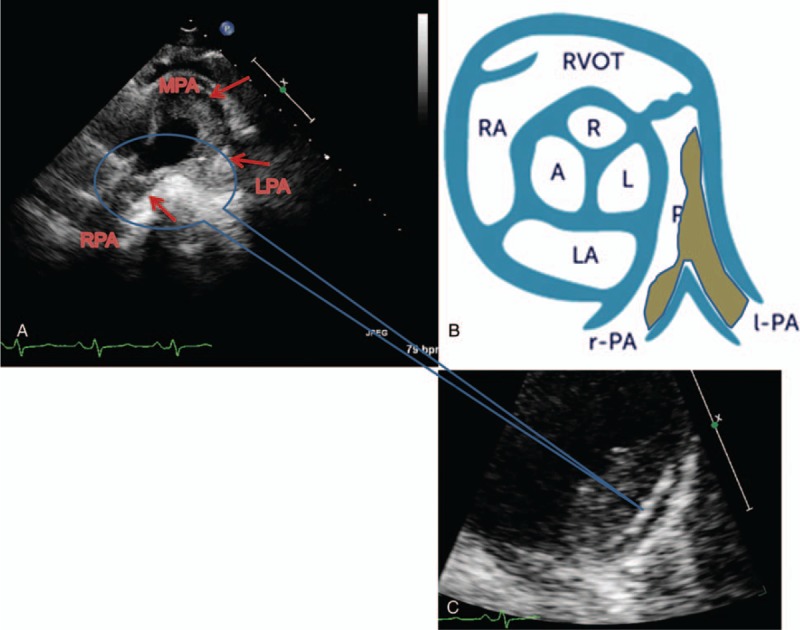
The presentation of ultrasonic cardiogram in pulmonary artery IgG4-RD causing PH. Ultrasonic cardiogram showed hypoecho lesion of lateral wall in main pulmonary artery (MPA) in patient 3, which extended to the left pulmonary artery (LPA), causing LPA occlusion. Hypoecho lesion extended to the right pulmonary artery (RPA), which induced severe RPA stenosis (A). Enlarge watching, the endothelium of hypoecho lesion was obviously thickening in pulmonary artery, and even destructive (C). The above presentation of ultrasonic cardiogram is like diagram as shown (B). IgG4-RD = IgG4-related disease, PH = pulmonary hypertension.

With the progression of diagnostic techniques, this disease has been recognized gradually, but the pathogenesis is not fully clear. According to previous study,^[2,10,20–24]^ some immune cells and cytokines were measured in our study, and it suggested that the lesion of pulmonary artery was formed in multiple processes. To be specific, the changes of Th2 cell, Treg cell, and cytokines support the probable mechanisms mentioned in previous studies (Fig. 5).^[2]^ The presentation of ultrasonic cardiogram also approved this probable IgG4-RD causing PH mechanism (Fig. 6).

In conclusion, to the best of our knowledge, these patients with IgG4-RD presenting pulmonary artery obstruction causing PH is rarely reported in previous studies. These diseases might be delayed or mistakenly diagnosed as pulmonary thrombus or malignant tumor. Therefore, a high index of awareness is required for the early diagnosis and treatment of this disease, which can prevent disabling tissue fibrosis, severe organ failure, and even death. The image characteristics of PET/CT might be valuable clues for the early diagnostic accuracy of this disease.

## Author contributions

**Conceptualization:** Yong Wang.

**Data curation:** Hui Deng, Sheng Zhao, Yunlong Yue, Yong Liu, Yali Xu, Jin Qian, Xiaorong Ma, Peiliang Gao, Xiaoyan Yao, Xin Jiang, Xiqi Xu, Zhicheng Jing, Yong Wang, Lei Pan.

**Formal analysis:** Xinying Xue.

**Funding acquisition:** Xinying Xue.

**Investigation:** Sheng Zhao, Zhicheng Jing, Lei Pan.

**Methodology:** Sheng Zhao, Yong Liu, Zhicheng Jing, Lei Pan.

**Project administration:** Yunlong Yue, Yong Liu.

**Resources:** Yunlong Yue, Yong Liu, Yali Xu, Jin Qian, Xiaorong Ma, Xiaoyan Yao, Xin Jiang, Yong Wang.

**Software:** Yunlong Yue, Yong Liu.

**Supervision:** Zhicheng Jing, Yong Wang, Lei Pan.

**Writing – original draft:** Peiliang Gao, Lei Pan, Xinying Xue.

**Writing – review & editing:** Sheng Zhao, Lei Pan, Xinying Xue.
